# Health professionals' perspectives of integrating meditation into cardiovascular care: A descriptive qualitative study

**DOI:** 10.1111/hsc.13849

**Published:** 2022-05-25

**Authors:** Angela Rao, Michelle DiGiacomo, Jane L. Phillips, Louise D. Hickman

**Affiliations:** ^1^ Improving Palliative, Aged and Chronic Care through Clinical Research and Translation (IMPACCT), Faculty of Health University of Technology Sydney Sydney New South Wales Australia; ^2^ School of Nursing Queensland University of Technology Kelvin Grove Queensland Australia; ^3^ University of Wollongong Wollongong New South Wales Australia

**Keywords:** cardiac rehabilitation, meditation, mental health, qualitative research, self‐management

## Abstract

Preliminary research suggests that meditation may provide benefits in psychological health and well‐being in people with cardiovascular disease (CVD). However, little is known about health professionals' perceptions of the barriers and facilitators to integrating meditation into CVD. A descriptive qualitative study design with semi‐structured interviews was used to explore the acceptability of integrating meditation into outpatient CVD programs and the organisational factors that may affect its integration. Clinicians were recruited through purposive and snowball sampling. E‐mail addresses were obtained from publicly listed profiles of cardiovascular and relevant health organisations. Interview questions included perspectives of organising or delivering meditation within a health setting, format of meditation delivery, organisational or other factors that facilitate or present barriers to integrating meditation into clinical practice, and perceived risks associated with integrating meditation in clinical settings. Verbatim transcripts were thematically analysed using an inductive approach and the Braun and Clarke (2006) method to identify themes within barriers and facilitators to implementation. Eighteen predominately female (61%) senior nursing and medical professionals (61%), as well as health managers (17%), psychologists (11%) and allied health professionals (11%), aged 40–60 years were interviewed between 18 May 2017 and 29 March 2018 in Australia via telephone, or face‐to‐face at a university or the participants' workplace. Three key themes were identified including: enhancing awareness of meditation within a biomedical model of care, building the evidence for meditation in CVD and finding an organisational fit for meditation in cardiovascular care. Meditation was perceived to sit outside the existing health service structure, which prioritised the delivery of medical care. Health professionals perceived that some physicians did not recognise the potential for meditation to improve cardiovascular outcomes while others acknowledged meditation's positive benefits as a safe, low‐cost strategy. The benefits of meditation were perceived as subjective, based on preliminary evidence. Health professionals perceived that aligning meditation with health organisational objectives and integrating meditation into outpatient cardiac rehabilitation and community‐based secondary prevention pathways is needed. A fully powered clinical trial is required to strengthen the evidence regarding the role of meditation for psychological health in CVD. Generating clinician engagement and support is necessary to enhance awareness of meditation's use in cardiovascular secondary prevention.

**What is known about the topic?**

- Depression and anxiety are under‐recognised and under‐treated in people with established CVD.
- Mental disorders reduce the ability of people with cardiovascular disease to adhere to their medications and recommended lifestyle changes, and influences cardiovascular risk.
- Meditation is one non‐pharmacological intervention that may reduce the prevalence and severity of depression, anxiety and stress symptoms in people who have experienced a cardiac event.

**What this paper adds**

- Some clinicians did not recognise the potential of meditation as a novel secondary prevention strategy for people with CVD, while others supported its potential as a low‐cost strategy for mental health and well‐being in people requiring additional support.
- Outpatient cardiac rehabilitation and community‐based programs were identified as ideal settings for the integration of meditation techniques as a component of stress‐management.
- Awareness of current evidence and the generation of definitive evidence from a well‐designed multicentre randomised controlled trial of meditation is needed to enable health professionals to successfully integrate meditation into routine cardiovascular secondary prevention programs.

## 1 INTRODUCTION

CVD remains the leading causes of death globally, accounting for 17.9 million deaths per year (Mendis et al., 2011). Depression prevalence in CVD is at least doubled when compared with the general population (Lichtman et al., 2008) and moderate depressive symptoms occur in approximately 15% to 18% of cases (Colquhoun et al., 2013; Rao et al., 2020). Mental disorders that result in significant functional impairment and reduce people's use of healthcare services are now considered as influencing CVD risk (Pedersen et al., 2017; Visseren et al., 2021).

While most people with CVD have modifiable risk factors, few countries have successfully implemented cardiovascular preventative strategies that changes people's lifestyle and health behaviours (Piepoli et al., 2016). National (Chew et al., 2016) and international guidelines (Visseren et al., 2021) recommend that people with established CVD and co‐morbid mental disorders require intensive attention and support to promote adherence to lifestyle changes and treatment, including referral to a structured secondary prevention service. In Australia, cardiac rehabilitation programs (CR) are provided both in hospital (phase 1), shortly after discharge from hospital (phase 2) or longer term delivery of preventive services (phase 3 or 4). Outpatient CR programs are classified as ‘hospital related services’ that occur following a previous hospital admission, and are predominately funded by respective state governments using an activity‐based funding (ABF) model. In an ABF model, hospitals get paid according to the number of patients they treat, as well as the complexity of patient care (Australian Government, 2020).

The premise of CR programs is to facilitate improvement in baseline functioning and to promote self‐management, which is inclusive of psychological health and functional capacity (Woodruffe et al., 2015). However, between only one quarter and one half of eligible patients attend CR (Astley et al., 2020; British Heart Foundation, 2015), one quarter to one half of depressed or anxious patients do not complete these programs (Rao et al., 2020), and many secondary prevention programs do not include psychological support strategies beyond depression screening and GP referral to promote self‐management of cardiac symptoms.

Cardiovascular clinicians are well placed to support referral and access to non‐pharmacological strategies including Cognitive Behavioural Therapy (Gulliksson et al., 2011), collaborative care (e.g. social work case management, education about depression and anxiety and collaboration with a psychiatrist who initiates recommendations for treatment and referral according to patient preferences) (Huffman et al., 2014) and physical activity or exercise to enable people with CVD to optimise their psychological health and quality of life. However, to date, while trials to reduce depression in CVD have shown that prognosis improves as depression improves, significant differences in cardiac outcomes have not been established (Carney & Freedland, 2017).

The recent American Heart Association Meditation and Cardiovascular Risk Reduction scientific statement lends support to the use of meditation as a novel approach to the secondary prevention of CVD (Levine et al., 2017). Meditation can be defined as ‘a family of mental training practices aimed at monitoring and regulating attention, perception, emotion and physiology’ (Fox & Cahn, 2017, p. 4). Mindfulness meditation (Baer, 2014; Chambers et al., 2009) promotes present moment, non‐judgemental awareness of thoughts, feelings and sensations, without attachment to these thoughts, while concentrative meditation types, such as Transcendental Meditation (Alexander et al., 1987; Ooi et al., 2017), Benson's Relaxation Response (Benson et al., 1974) or guided imagery (León‐Pizarro et al., 2007), involve a focus on a specific mental or sensory activity, such as a repeated sound, visualised image or specific body sensations such as breath. Methods used to elicit state changes differ across practices, however both approaches can mutually influence and enhance each other, producing similar changes towards an expanded awareness or consciousness (Cahn & Polich, 2006; Lutz et al., 2008). Meditation may enable adaptive self‐regulation of emotional responses, by allowing individuals to gain reflexive awareness of implicit mental contents which eases transformation of cognitive and emotional habits (Lutz et al., 2008). While understanding of the biological mechanisms of action remain unclear, evidence from randomised controlled trials suggest that meditation may improve depression and anxiety (Nyklíček et al., 2014; Parswani et al., 2013; Rao et al., 2019), hypertension (Parswani et al., 2013) and quality of life (Chang et al., 2005; Curiati et al., 2005) in people with CVD, and may improve adherence to secondary prevention programs (Rao, 2020). However, there is little evidence of how to effectively integrate meditation into routine cardiovascular care.

The World Health Organization Innovative Care for Chronic Conditions Framework (World Health Organization, 2002) provides a theoretical framework for the consideration of contextual factors that impact on the capacity of an organisation to integrate mediation into cardiovascular care. Exploring the perceptions of health professionals involved in the prevention and management of CVD is necessary to understand the health organisational and health systems factors (Damschroder et al., 2009) that impact the role and integration of meditation into secondary prevention programs. It may also shed light on the potential to incorporate recommendations from international guidelines (Levine et al., 2017) that support the development of meditation research and practice within CVD outpatient programs for patients seeking additional psychological support.

### 1.1 Aims

The aim of the study was to investigate health professionals' perceptions of integrating meditation into secondary prevention cardiovascular care.

## 2 METHODS

### 2.1 Study design

This study used a descriptive qualitative study design (Colorafi & Evans, 2016), and semi‐structured interviews were undertaken. Reporting of this study adhered to the Consolidated Criteria for Reporting Qualitative Research (COREQ) checklist (Tong et al., 2007).

### 2.2 Participant selection

Participants were recruited through purposive and snowball sampling (Creswell & Plano Clark, 2018; Sadler et al., 2010). Purposive sampling allowed the researcher (AR) to intentionally select participants with experience with the key concepts being explored (Creswell & Plano Clark, 2007). Health professional e‐mail addresses obtained from publicly listed profiles of health professionals through relevant cardiovascular conference websites, relevant health organisations and the New South Wales/Australian Capital Territory Cardiac Rehabilitation directory (National Heart Foundation of Australia, 2015) ensured accessibility to relevant health professionals and recruitment feasibility (Sadler et al., 2010). This included an online search of e‐mails for keynote speakers at the 2017 Australian Cardiac Rehabilitation Association Conference, Perth, the 2017 Heart Foundation Women's Health Forum, Sydney and the 2017 Cardiac Rehabilitation Association Conference. Recommendations for other health professionals were obtained during the snowball sampling process, and in some instances, as a consequence of the snowball process direct e‐mail contact was given for a relevant health professional to the researcher.

The disadvantage, or potential bias of undertaking a snowball recruitment strategy could be an overrepresentation of participants of similar characteristics (Sadler et al., 2010). To counter this, a list of all participants screened and recruited was created to enable visual representation of the sample, gender, age range and professional roles. This was viewed by the researcher (AR) and a member of the research team (LDH) to ensure that a diversity of participants from all levels of cardiovascular health were included.

Participants were included if they were nursing, medical or allied health professionals working in cardiovascular clinical settings or managers or executives of relevant health organisations, and were willing to give informed consent and be interviewed. Participants were informed of the purpose of the research. The participants were not known to the researcher prior to the interview, except for one participant who was involved in a previous research project.

### 2.3 Setting and participants

Interviews were conducted individually face‐to‐face or via a Polycom telephone at a large university in Sydney, a private area of the participants' workplaces, or at a CVD conference venue with no one else present. Data were collected between 18 May 2017 and 29 March 2018.

### 2.4 Ethical considerations

This study was approved by the Local Health District's Human Research Ethics Committee and at the participating university. A description of the study was e‐mailed to interested participants, and formal written consent was obtained in person or a scanned signed copy of the consent form was sent to the researcher (AR) via e‐mail prior to the interview. Voluntary participation was emphasised, including participants' freedom to withdraw at any time, without any implications. No incentives were offered to encourage participation.

### 2.5 Data collection

**FIGURE 1:**
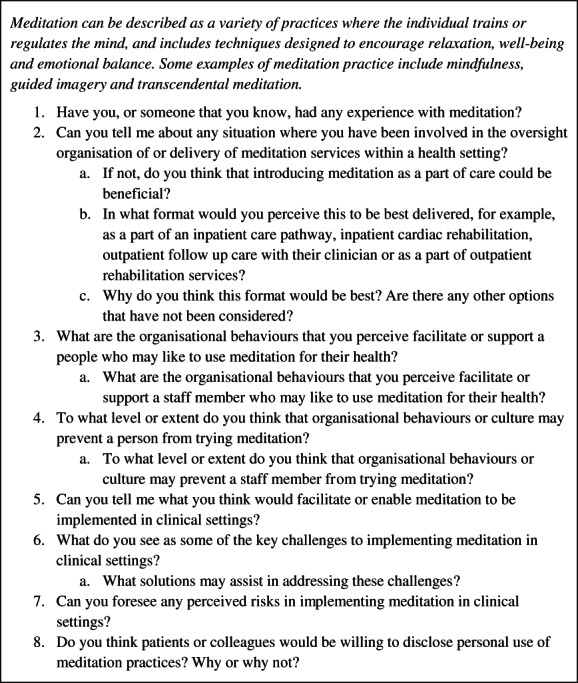
Health professional interviews: Semi‐structured interview guide

Semi‐structured interviews were used to generate in‐depth responses to pre‐set open‐ended questions (Jamshed, 2014). The majority of interviews were conducted by the researcher (AR). One interview was conducted by an experienced qualitative research team member (MD) to avoid potential for social desirability bias given the participant and AR knew each other. Interview guides were used to ensure consistency in issues explored (refer Figure 1) (Jamshed, 2014). The interview guide was informed by gaps identified in the literature (Rao et al., 2019), and previous research on barriers and facilitators to meditation in clinical care (Crane & Kuyken, 2013; Joyce et al., 2010). This interview guide was pilot tested on one participant, and was considered acceptable without modification. The researcher (AR) refined the interview technique with the research team (MD, LDH) after the completion of the first three interviews, which ensured consistency between interviews and interviewers, and ensured that neutrality in tone, posture and language was maintained (AR & MD). The interview sessions were of 20–60 min duration, unless more time was needed as determined by the participant. Reflexive accounts of the interviews were documented at the completion of each interview. These accounts contained notes on the tone of the interview, nuances made with respect to different topics and contextual details such as the dynamic between the researcher and the participant within the interview setting. Reflexive accounts were referred to during the process of transcription and data analysis to ensure accurate interpretation and representation of the data. Data collection and analysis were an iterative process that occurred concurrently such that the research team could establish what was known and areas where further depth was required (Morse et al., 2002). Data collection ceased when it became clear that no new information was being elicited and data saturation was achieved.

### 2.6 Data analysis

The Braun and Clarke (2006) method of thematic analysis was used in a series of six steps. An inductive approach was used to ensure that the themes generated were purely derived from the data (Thorne, 2000). All interviews were audio recorded and transcribed verbatim by a professional transcription service. These transcripts were read by the researcher (AR) along with corresponding voice recordings to ensure accuracy of the data. The digital files were de‐identified following transcription, and were coded using NVivo software version 11.

Data were coded by the researcher (AR) into categories without fitting into a pre‐existing coding frame or researcher preconceptions (Braun & Clarke, 2006). Codes generated by the researcher were then formatted into tables. This enabled the selection of the most relevant quotes within each code and ordered structuring of each quote so that the code could be combined with other codes prior to forming an overarching theme. Categories that were identified as potential themes were refined to ensure that the collated quotes within each category fit and the potential theme formed a coherent pattern. Subcategories that did not contain enough coherent data were broken down and fit into separate themes, set aside or discarded. Alternatively, subcategories were collapsed into a larger existing or new potential theme to synthesise and better reflect the key ideas contained within the data, and to ensure coherence and identifiable distinctions between central concepts (Braun & Clarke, 2006). The remaining potential themes were refined for coherence. Data were further refined to reflect themes and subthemes that were to be presented in the analysis. Ongoing reflexive dialogue occurred between the researchers throughout the process of data collection, coding and analysis to ensure rigour and consensus was reached.

## 3 RESULTS

Of the 48 eligible participants contacted, 18 health professionals consented to be interviewed. The average duration of each interview was 49.6 (*SD* ± 19.5) minutes. Participants were predominately female (*n* = 11; male *n* = 7) and aged between approximately 40 and 60 years (refer Table 1). All participants were experienced clinicians (i.e. allied health professionals (AH), medical doctors (MD), registered nurses (RN), psychologists (P), health managers (HM)) or health researchers and all held senior roles within their departments. Reasons for non‐participation included non‐respondents (*n* = 22), no response after initial interest (*n* = 1), scheduling difficulties (*n* = 4), time constraints (*n* = 2) or the topic was considered to be outside their area of specialisation (*n* = 1).

**TABLE 1:** Participant characteristics

Characteristic	*N*	%
Gender		
Male	7	38.9
Female	11	61.1
Clinical background		
Allied health (AH)	2	11.1
Medical doctor (MD)	5	27.8
Registered Nurses (RN)	6	33.3
Psychologist (P)	2	11.1
Health management (HM)	3	16.7
Location		
Australia (urban)	13	72.2
Australia (rural)	2	11.0
United States	1	5.6
Sweden	1	5.6
Denmark	1	5.6

Three key themes were generated from the data: (i) Enhancing awareness of meditation within a biomedical model of care; (ii) Building the evidence for meditation in CVD; and (iii) Finding an organisational fit for meditation in cardiovascular care, and are discussed along with relevant supporting quotes in the following section.

### 3.1 Enhancing awareness of meditation within a biomedical model of care

Meditation was perceived by participants to sit outside the existing health service structure that prioritises the delivery of medical care. Participants suggested that the current siloed healthcare system reflected the historical and deeply embedded biomedical disease model of care.

…but I think you know, it is about the system and the way it has evolved. It has become so siloed. You know the fact that we're siloed between the physical health streams, to then take that leap and join up physical and mental health is another. You know, it is a big piece of work. Doesn't mean it shouldn't. You know, it's got to happen (HM, Participant 002).

Consequently, the current healthcare structure was not geared to adequately identify and address the psychological support needs of increasingly complex patients, like people with CVD. It was suggested that clinicians driving cardiovascular care required awareness of the link between psychological health and cardiovascular outcomes.

Not so much I think because that they didn't see the importance, but it was more that they didn't understand the importance (of meditation), and it really comes out of their training…. But I see that changing (HM, Participant 001).

Many suggested that the use of meditation for self‐management support was largely perceived to be outside the scope of usual clinical care. The unintended consequence of conventional medical training was that it limited some clinicians' ability to conceptualise meditation as an integrated element of self‐care. Participant's suggested that there was vested interest in maintaining the structure of health service delivery for the promotion of medical care, which was not inclusive of meditation.

You know, I definitely think there is a part of the medical community that sort of disregards that area (meditation) because it's non‐pharmacological, which is sort of sad, but that's the reality…so you know I think sort of the barriers are convincing clinicians that these interventions do help the patient (MD, Participant 012).

Some participants referred to their perceptions of the limited available resources to support the integration of non‐pharmacological therapies, including meditation, which were prioritised for the delivery of medical and surgical interventions.

Given this reality, there was still quite a long way to go to convince health organisations that meditation had a primary and secondary role to play in CVD.

### 3.2 Building the evidence for meditation in CVD

Participants' were divided on the value and role of meditation, which was largely due to their perceptions of the level of evidence and the relative importance that evidence plays in patient decision‐making.

Some participants perceived that meditation had a role to play in CVD secondary prevention because of its positive effects and that it was considered a low‐cost and relatively safe non‐pharmacological strategy.

…many people attribute their heart attack or heart disease to stress… So people are actually quite receptive to a (meditation) technique. And you know there are a lot of non‐pharmacological techniques where the evidence is not very strong but the uptake is high (RN, Participant 005).

Others considered the perceived benefits derived from meditation to be subjective, as exemplified in this quote.

If they think it would be of benefit to them, who am I to say that it won't? It's a bit like when patients come to me with ‘natural therapies’. I know nothing about what they are doing with their ‘natural therapies’ and I say “As long as you don't stop taking my pills”… (MD, Participant 008).

One participant doubted that meditation could tangibly improve patient outcomes, and was uncertain as to whether promoting meditation was a clinical responsibility. This participant implied that the effects of meditation are related to a placebo effect, and perceived that meditation had a relatively minimal, if any clinical benefit compared with conventional cardiac medications and interventions.

Other participants were more likely to endorse and implement mindfulness techniques due to greater perceived acceptability and the widespread adaptation of mindfulness‐based practices to Western clinical settings. However, some participants were more hesitant.

…I would hypothesize that it (meditation) can help people be better able to regulate their emotional reactions to situations. So it improves emotion regulation. It clearly helps to evoke the relaxation response in the body…However, I can't say that I absolutely know for sure that I know that is the situation (P, Participant 018).

These participants were reluctant to make definitive conclusions about meditation's efficacy, which created ambivalence about the role of meditation in clinical practice.

#### 3.2.1 Perceived lack of evidence

Many participants were not ready to openly endorse the broad implementation of meditation practices in clinical care because they perceived that meditation is not underpinned by high level evidence. Additionally, some participants were uncertain about the level of the existing evidence.

So if you want it (meditation to be) funded by government, you need really good evidence that it does something……the amount of evidence that meditation actually makes a difference is pretty low (MD, Participant 003).

The perception of a lack of evidence to support prioritisation of meditation within clinical care leads it to being devalued as a primary or secondary prevention strategy. This perception was both enhanced and driven by government funding bodies which did not prioritise meditation research and practice.

…What evidence can you show us that if we change things, things will improve for either the patients or the profession? Data, give us data, that's the other thing they will want. Evidence. And without that, you can't move (HM, Participant 002).

As participants operated within a culture of evidence‐based practice, all their clinical decisions were informed by the available evidence, patient preference and their own clinical experience. To translate meditation research into practice, participants suggested that effectiveness and efficacy data were required to change their practices. The above excerpt implies that self‐report of positive meditation outcomes in clinical research was perceived to be less definitive.

### 3.3 Finding an organisational fit for meditation in cardiovascular care

Successfully navigating organisational change was required to find a fit for meditation within the current structure of health service delivery. The key facilitators for meditation were identified as subthemes, including (i) *driving organisational change*, which described the need to align with health organisational objectives; (ii) *integrating meditation into secondary prevention pathways* to create opportunities for meditation programs; and (iii) *accreditation or training to facilitate meditation referral*.

#### 3.3.1 Driving organisational change

Successfully influencing health executives by operating within the health organisational structure was required to create a fit for mediation.

I think the best funding source there would be, in the area of mental health, which is a very, very popular area funding at the moment in the current landscape…I think there is a lot of opportunity from government to get funding because it will increase productivity in the workplace. And reduce hospital admission times and all sorts of things. So the government will be approachable (MD, Participant 012).

Participants suggested that a multi‐strategy approach was required to generate health organisational support for the integration of meditation programs into existing services. These strategies included: identifying mutually beneficial outcomes between the meditation service provider and the relevant health organisation, such as couching meditation within state government mandated key performance indicators such as patient experience, readmissions and quality of life, and promoting meditation within a person‐centred model of care or for mental health. Participants implied that health executives could be motivated to support meditation as a part of addressing key performance indicators and had some flexibility as to what strategies were implemented to address this process.

Participants generally considered the need to justify health organisation resource allocation to establish meditation programs. These participants perceived that demonstrating the potential for meditation programs to reduce health expenditure was required to generate executive support for meditation.

… And obviously if there's (Activity‐based funding) attached to that then that strengthens the case. In the public health system it's the way the hospital gets income from the government for the work that it's doing and the services that it's providing for patients (AH, Participant 015).

Understanding the language and internal processes of health organisational operating systems for resource allocation were important facilitators to leveraging health organisational resources. Sustainability of meditation programs and increased funding for the organisation through numbers of patients serviced were strong incentives for health organisational change.

#### 3.3.2 Integrating meditation into secondary prevention pathways

Cardiac rehabilitation (CR) was identified by the majority of participants as the best fit for the delivery of meditation programs within the cardiovascular care trajectory. The creation of secondary prevention pathways for meditation was perceived to better enable people with CVD to become aware of and access meditation within CR programs.

…And I thought it was interesting because it (survey) doesn't look at meditation, but it talks about stress management. And it's (stress management) actually a part of the cardiac rehab program…what strategies they used for that I don't know, but, you know that there's a place for it in there… (HM, Participant 002).

Participants identified the potential to incorporate meditation within the stress‐management component of CR service delivery. However, the extent to which stress‐management techniques were applied across different clinical settings was unknown, and implementation was left to the discretion of the health professional. Other participants focused more on the potential role of meditation for depression in CR programs.

Many participants also identified that there was no existing pathway for referral for meditation programs within CVD secondary prevention.

But I think you need to streamline the path of entry. I don't know where you would refer patients for this sort of thing. And I'll be really frank with you. If I don't, the chances are that a lot of people don't… (MD, Participant 008).

Reduced prioritisation of non‐pharmacological approaches to CVD secondary prevention, including CR, was perceived to underpin the lack of existing referral pathways for meditation within cardiovascular care.

I mean it's not something that perhaps is considered a core component of cardiac rehabilitation‐ but yeah it probably is seen as, you know, perhaps a bit of an add‐on…. (AH, Participant 015).

While the majority of participants supported the introduction of a referral pathway for meditation, lack of inclusion of meditation within CR guidelines was perceived to drive health professional reluctance to refer patients to meditation.

Opportunities for community referral were also discussed.

So I think getting support across the health care team, particularly I think within general practice is something to think about as well. Or just letting them know about the techniques and the purpose might be useful (RN, Participant 005).

Participants identified the appropriateness of community referral to meditation programs due to an increasing emphasis on outpatient services and continuity of care.

…things are moving towards that rehabilitation will be more implemented in a municipal setting. …the university medical centres, they're becoming more highly specialized for the complex patients, and then things are moving out towards the GPs… (P, Participant 007).

Increasing burden on the health system due to increasing patient acuity was perceived to underpin health professionals' support for meditation within community‐based stress‐management programs

#### 3.3.3 Accreditation or training to facilitate meditation referral

Participants' perspectives on formal accreditation and training were divided into two groups: those who recognised the role of formal accreditation and training in facilitating referral to meditation; and those who perceived that health professional experience in meditation practices was sufficient to ensure competence in delivery.

I would suggest that we would be more comfortable if they had some form of credentialing. You know, if someone, if a service provider sort of came on recommendation and they didn't have credentialing then that might be acceptable. But you know if they had neither and we really didn't know much about the veracity of the service they provide, we would probably be a little reticent to refer. And that's fairly consistent with referral to other service providers as well (AH, Participant 015).

Meditation was perceived by many participants to be underdeveloped as a professional practice. The lack of widespread formal accreditation processes and responsibility to fulfil a duty of care underpinned the need for discernment when accepting referrals for meditation practitioners.

What constituted adequate training in meditation was also discussed.

…so I would say if someone's got their own regular practice that they've had for several months and they are willing to sort of follow a standardized protocol which we know is safe, then they are the best people to do it (P, Participant 018).

Some participants perceived that a formal qualification in meditation alone did not guarantee the necessary competence in implementing meditation practices, rather that mentorship and sustained personal meditation practice was needed. Protocols in clinical settings were favoured in order to maintain duty of care to the patient.

## 4 DISCUSSION

To our knowledge, this is the first Australian study to explore health professional perceptions of the organisational barriers and facilitators to implementing meditation in CVD. This study has highlighted the importance of strengthening meditation evidence and promoting understanding of the current evidence for meditation before proceeding to implementation. Despite meditation being reported in an American Heart Association scientific statement as a low‐risk adjunct (Levine et al., 2017), it is evident that awareness of substantive effectiveness evidence is needed to increase health professionals' support for meditation practices, which is consistent with previous systematic review findings (Goyal et al., 2014; Rao et al., 2019). These findings are also consistent with previous research that highlighted (1) funding, provider hesitancy to recommend meditation in the context of limited research and lack of familiarity with the meditation modality and facilitators (LeVasseur et al., 2019), and (2) the focus on acute presentation, politically initiated restructuring and budgetary constraints (Crane & Kuyken, 2013; Gibbons & Thomas, 2021).

In the United States, however, there are examples of the implementation of meditation into existing CR programs. A ‘meditation station’ has been set up in one program that includes the use of a Kindle and a 1–5 minute meditation using the Headspace app (Barton & Mikan, 2020). Information on how to use the app and information on the benefits of meditation are also provided. Guided meditation videos are provided online as part of a stress, relaxation and resilience component of another CR program (The General Hospital Corporation, n.d), which are freely available. Meditation has also been implemented into a multicomponent nurse‐led 13‐week CR program as part of a variety of techniques (yoga, breath focus, mindfulness and visualisation) used to elicit the relaxation response. Discussion sessions related to stress‐management included recognition of the warning signs of stress and use of cognitive restructuring skills to challenge thinking patterns and patients were asked to write a weekly cognitive journal (Casey et al., 2009). The Cleveland Clinic Lerner College of Medicine has also integrated meditation and mindfulness into third year medical training to increase awareness of meditation among future cardiologists and other medical professionals (Calabrese, 2018).

*Implications for practice*: Current findings demonstrate that most health professionals considered meditation as a useful self‐management strategy that supported behaviour change and were open to exploring future meditation research and practice for people requiring additional psychological support or for general health and well‐being. Affiliating with outpatient cardiac rehabilitation program providers and referral to community‐based health professionals, such as psychologists, psychotherapists or general practitioners will be helpful to integrate meditation as an additional stress‐management technique (Gallagher et al., 2020; Pedersen et al., 2017). This may be achievable by structuring and embedding quality improvement projects within the organisational fabric, building a shared understanding and commitment to quality improvement, and developing formal and informal opportunities for learning (Crane & Kuyken, 2013).

Provider incentives are required for health organisations to promote the integration of innovative strategies to support self‐management (World Health Organization, 2002). To find a fit for meditation within current health service models, health professionals and meditation practitioners need to demonstrate that meditation addresses key performance indicators, such as improving patient experience and quality of life, and can be presented in a business model that evaluates cost‐effectiveness.

*Implications for research*: Working with relevant national cardiac rehabilitation organisations to promote meditation research and practice is essential to driving the case for meditation as a low‐cost, low‐risk, secondary prevention strategy. A Delphi study could be used to inform local and state‐wide meditation guideline development. For example, a modified Delphi study was recently used to endorse evidence‐based relaxation strategies (including meditation and mindfulness) to reduce client stress and anxiety within ‘clinical and community preventive service and health promotion’ physical therapy competency assessment (Magnusson et al., 2020). This research is essential to understanding how to best develop referral processes to support the integration of meditation within secondary prevention pathways, and to explore possibilities to integrate evidence‐based mind–body therapies within existing cardiac rehabilitation core components (Woodruffe et al., 2015).

### 4.1 Limitations

Limitations to this research include the use of purposive and convenience sampling approaches, which may introduce a self‐selection bias, thereby reducing generalisability of the study to the national CVD clinical population. Further research into CVD clientele perspectives of the barriers and facilitators to meditation use in CVD secondary prevention is also warranted. The researcher (AR) has experience in meditation, however was constantly aware of her perspective of meditation during the interview process, ensuring that neutrality in tone, posture and language was maintained. She presented as open to participants' perspectives during interviews and withheld her own point of view.

## 5 CONCLUSION

Health professional engagement and support is required to better establish the role of meditation in cardiovascular secondary prevention. A large, multi‐centre, randomised controlled trial is required to strengthen the evidence for meditation in CVD and to promote uptake, including the evaluation of cost‐effectiveness and sustainability.

## FUNDING INFORMATION

AR was supported by an Australian Government Research Training Program Scholarship from the Commonwealth Government and a University of Technology Sydney Research Excellence Scholarship.

## CONFLICT OF INTEREST

The authors declare that there are no conflicts of interest.

## AUTHOR CONTRIBUTIONS

AR, LDH and MD conceived and designed the project, and acquired the data. All authors were involved in the analysis and interpretation of the data. AR wrote the manuscript. All authors were involved in subsequent revisions of the manuscript. All authors have approved the submitted version. AR agreed to be accountable for all aspects of the work in ensuring that questions related to the accuracy or integrity of any part of the work are appropriately investigated and resolved.

## ETHICS APPROVAL STATEMENT

This study was approved by the Western Sydney Local Health District's Human Research Ethics Committee and ratified at the University of Technology Sydney.

## Data Availability

Not applicable.
